# Free flap for soft palate reconstruction: long-term functional evaluation of a new technique

**DOI:** 10.1007/s00405-021-06897-0

**Published:** 2021-06-02

**Authors:** A. Colliard, L. Pincet, C. Simon, L. May, K. Lambercy

**Affiliations:** 1 Department of Otorhinolaryngology and Head and Neck Surgery, Centre Hospitalier Universitaire Vaudois, Rue du Bugnon, Service d’ORL, BH 10 CHUV, Avenue du Bugnon, 46-1011 Lausanne, Switzerland; 2 Department of Maxillofacial Surgery, Centre Hospitalier Universitaire Vaudois, Rue du Bugnon, 46-1011 Lausanne, Switzerland

**Keywords:** Carcinoma, Squamous cell, Head and neck neoplasms, Soft palate, Reconstructive surgical procedure, Deglutition, Free flap, Microsurgical

## Abstract

**Purpose:**

The soft palate (SP) has a complex anatomy and physiology. Reconstruction after tumour resection is a challenge, and procedures that only restore bulk don’t give good results. We aim to present a new technique for the in-setting and the functional outcomes.

**Methods:**

We retrospectively included in a monocentric retrospective cohort study every patient with a first diagnosis of a soft palate squamous cell carcinoma (SPSCC), who underwent a tumoral resection with a free flap reconstruction, from February 2013 to July 2017. For the in-setting, a special care is given for the flap in-setting: we suture the flap more caudally than usual under the tongue base, creating a neo-posterior pilar. The primary outcome was the deglutition function, assessed by the M. D Anderson Dysphagia Inventory (MDADI). We also analyzed the patient’s quality of life with the FOSS score and the occurrence of nasal regurgitation or larynx aspiration.

**Results:**

We included twenty patients, with a median follow-up of 26.5 months. The median MDADI score was 89, and the mode was 93. A Fisher test shows a significant improvement of MDADI scores for unilateral vs bilateral reconstructions (*p* = 0.03). The median FOSS score was 2, and the mode was 2. Seven (35%) patients complained of nasal regurgitation, three (15%) reported episodic laryngeal aspiration.

**Supplementary Information:**

The online version contains supplementary material available at 10.1007/s00405-021-06897-0.

## Introduction

The soft palate has a complex anatomy and physiology. The function of the soft palate is mainly to separate the rhinopharynx and the oropharynx. During swallowing, the soft palate tenses and helps in pushing the food down the digestive tract. During nasal breathing, the soft palate depresses and gets in touch with the tongue’s root, making sure that the food doesn’t pass to the oropharynx and gets in the way of the stream of exhaled air. It protects the nasal passage by diverting a portion of the excreted substance to the mouth during sneezing.

Furthermore, the soft palate plays an essential role in speech, as it enables the pronunciation of velar consonants together with the tongue.

Therefore, reconstruction after tumour resection is a challenge for surgeons. While keeping an optimal preservation of cancer surveillance, it has to reproduce the soft palate and its velopharyngeal sphincter functions. [1, 2].

First, palatal reconstruction has to efficiently separate the nasal and oral cavities to ensure air and water tightness. But reconstructive techniques that only restore bulk do not give good results. Small defects are eligible for local reconstruction. It minimizes the sensory loss and preserve as possible coordinated dynamic functions [2]. On the contrary, large defects remain a surgical challenge. Many surgical techniques have been described, trying to reproduce the physiology while filling the defect.

Therefore, we aim to share our experience with free flaps reconstruction. Using well-known micro-anastomosed free-flaps, we developed a new technique for insetting. Functional outcomes through questionaries enabled the evaluation of this technique.

## Materials and methods

### Patients

We retrospectively included in a monocentric retrospective cohort study every patient with a first diagnosis of a soft palate squamous cell carcinoma (SPSCC), who underwent a tumoral resection with a free flap reconstruction, from February 2013 to July 2017.

We excluded every patient who underwent local or regional flaps. To focus only on the surgery’s functional result, we excluded the patients presenting a tumour recurrence during the study. Preoperative patient’s and tumour’s characteristics were analyzed. We classified surgical complications following the Clavian-Dindo classification [3].

The primary outcome was the deglutition function, assessed by the M. D Anderson Dysphagia Inventory (MDADI) [4]. We also analyzed the patient's quality of life with the FOSS score [5] and the occurrence of nasal regurgitation or larynx aspiration. For that, we asked simple questions, such as “Do you have nasal regurgitation?”, “Dou you have nasal regurgitation”, and “Yes” or “No” answers were collected. Responses were obtained from June 2017 to March 2019 during a follow-up consultation. A clinical examination, including a pharyngo-laryngoscopy, allowed to get photo-documentation.

We present in the results descriptive analysis. Because we have a small sample of patients, we did the statistical analysis with Fisher-tests.

All surgeries were performed by the same ENT and maxillo-facial senior surgeons. All patients signed an informed consent for this publication.

The Clavian-Dido classification, the MD Anderson Dysphagia Inventory and the FOSS score are available in the supplementary material.

### Surgical strategy

We perform a free-flap reconstruction for every patient with a lesion larger than 4 cm. We use an Allen test for defining the strategy. We prefer to use the antebrachial (AB) flap, but we can make anterolateral thigh (ALT) flaps in case of a negative Allen test. Both can be used without size limitations. If an osteocutaneous reconstruction is needed, we perform peroneal free-flap (P). For each flap, we provide a cutaneous palette a little longer than the anticipated defect (2–3 cm longer).

Flaps will swallow in the first days after the surgery. Therefore, we provide at the beginning of the surgery a temporary tracheostomy for each patient. Moreover, it helps to share the upper aerodigestive tract with the anesthesiologists. A nasogastric tube is also placed in the beginning.

Either transorally or transmaxillary approach can be chosen, depending on the tumour’s accessibility. All patients undergo a selective uni or bilateral neck dissection according to the international recommendations [6].

**Fig. 1:**
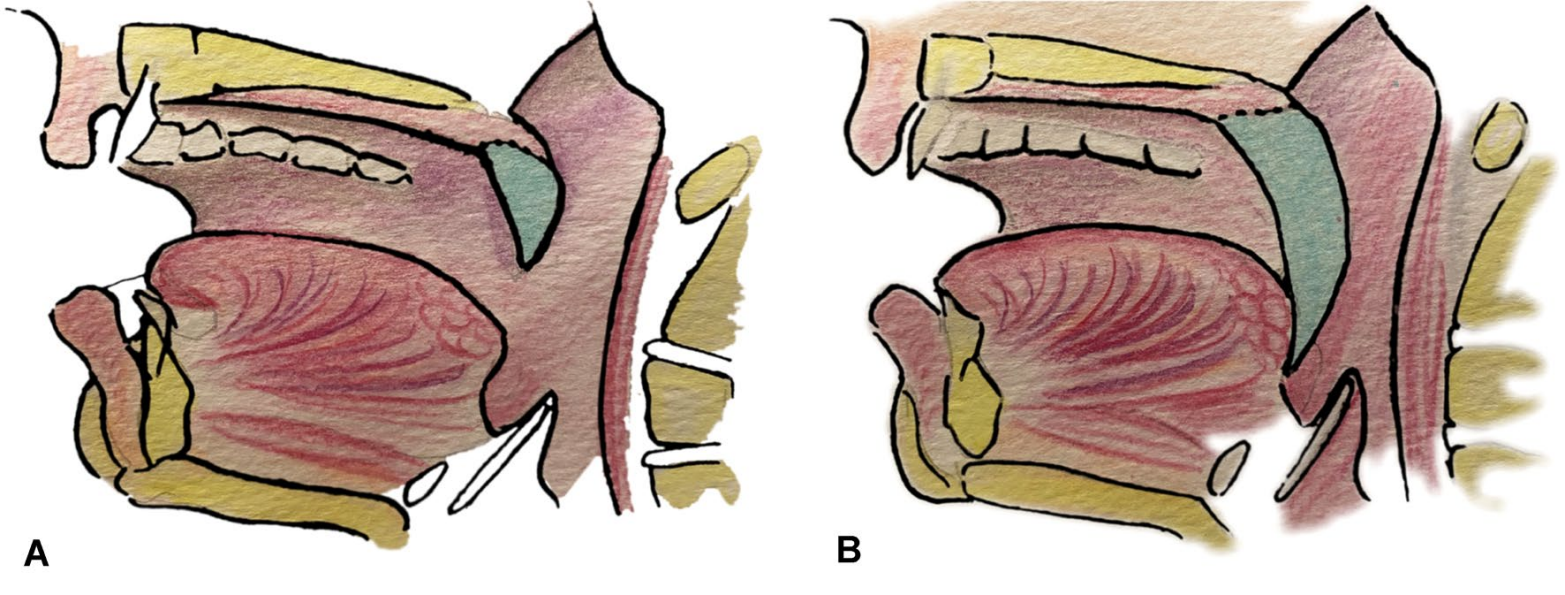
**A** Regular Flap In-setting. **B** New technique for the Flap In-setting

**Fig. 2:**
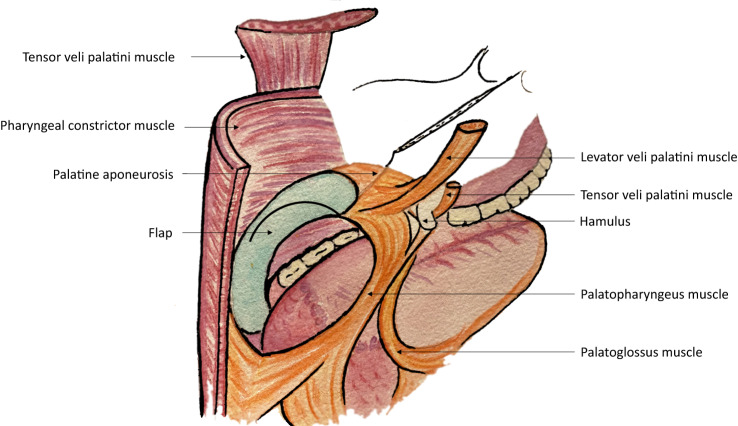
Postero-lateral view of the Flap In-setting

**Fig. 3:**
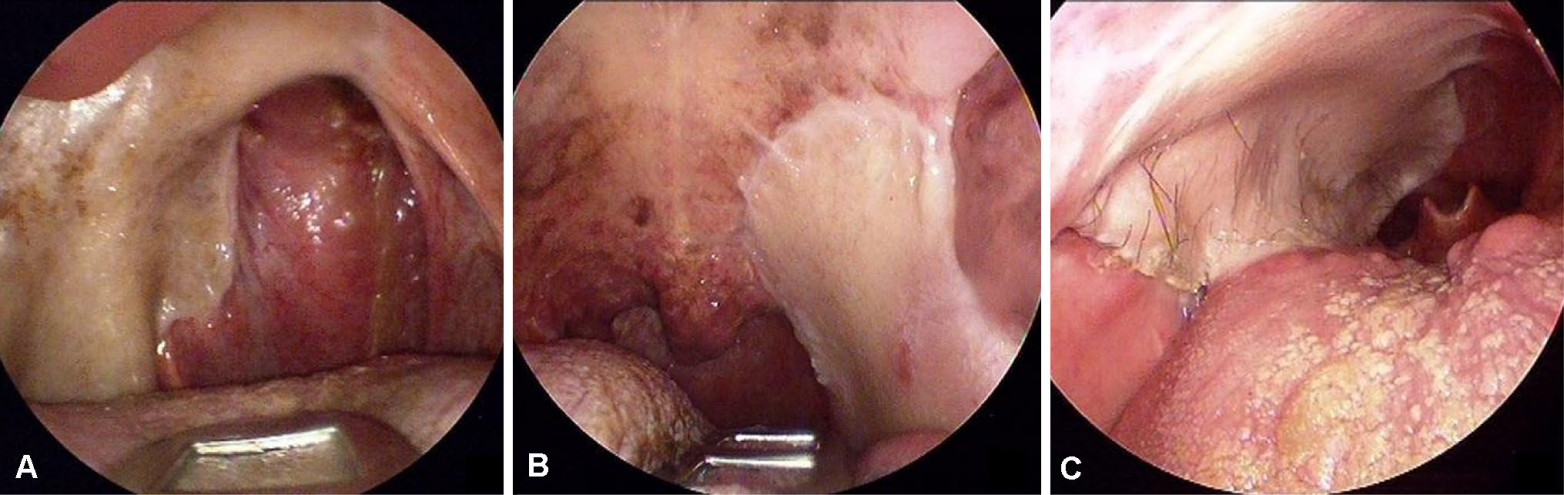
**A** Right flap, frontal view. **B** Left flap, frontal view. **C** Right flap, view of the oropharynx. The flap is placed lower than usual under the tongue base

The particularity of our technique relies on the in-setting:

We give special care to suture the flap more caudally than usual under the tongue base (Fig. 1). Sutures will be submitted to important tractions during swallowing or sneezing. Therefore, we recommend performing reliable sutures, particularly in the tongue base and near the maxillary tuberosity. We usually use closely spaced Donati stitches. The extra centimetres of the cutaneous palette provided in the beginning will help to give some flexibility. However, we want to keep a light tension. Once integrated, the flap has to form a neo-pilar (Fig. 2). It restores part of the original anatomy of the soft palate. Figure 3 illustrates the result after 1 year.

In postoperative care, the cannula is usually closed after 7 days and the tracheostomy sealed following a standard medical procedure.

Oral refeeding is allowed after 1 week. In case of insufficient oral intake after 3 weeks, a temporary gastrostomy is discussed with the patient.

## Results

Twenty-five patients underwent soft palate reconstruction during this period in our centre. We excluded two patients who died during the study and three for tumoral recurrence. Recurrences were revealed by systematic follow-up imaging (MRI or PET-CT). Finally, 20 patients were included.

Patients’ characteristics and surgical results are shown in Table 1. Statistical analysis is detailed in Table 2.

**Table 1:** Patients’ characteristics and results

Patients	Age	Sex	Tumour primary site	TNM	Soft palate reconstruction^a^	Flap type^b^	Surgical complications^c^	Approach^d^	Adjuvant treatment^e^	PEG	Tracheotomy	FOSS score	MDADI score	Nasal reflux	Larynx aspiration
1	72	M	Oropharynx	pT2pN0M0R0	B	AB	3	TO	0	No	Yes	2	90	0	0
2	70	M	Oropharynx	pT2pN0M0R0^f^	U	AB	0	TO	RT	No	Yes	2	93	1	0
3	74	M	Oropharynx	pT2ypN0Pn1M0R0	B	AB	0	TO	RCT	No	s/p LT	2	92	1	0
4	60	M	Oropharynx	pT2pN0M0R0^f^	B	AB	3	TM	RCT	No	Yes	5	41	1	1
5	83	M	Oropharynx	pT2pN2bM0R0	U	ALT	0	TM	RCT	No	Yes	2	84	0	0
6	67	M	Oropharynx	pT2pN0M0R0	U	AB	3	TM	0	No	Yes	1	99	0	0
7	58	M	Oral cavity	pT2pN1M0R0^f^	B	AB	3	TO	RT	No	Yes	2	90	0	0
8	66	M	Oral cavity	pT1pN0M0R0	U	AB	0	TO	0	No	Yes	1	93	0	0
9	65	M	Oral cavity	ypT2ypN0M0R0^f^	U	ALT	3	TO	3	No	Yes	2	88	0	0
10	61	M	Oropharynx	pT3pN2bM0R0	U	AB	2	TO	RCT	Yes	Yes	1	86	0	1
11	42	M	Oral cavity	pT4apN1M0R0	U	P	3	TM	RCT	No	Yes	2	92	1	0
12	67	M	Oral cavity	pT3pN2bM0R0	U	AB	3	TM	RT	No	Yes	2	95	0	0
13	61	M	Oral cavity	pT3pN2bM0R0	U	P	3	TM	RCT	Yes	Yes	5	66	1	0
14	66	F	Oropharynx	pT1pN3M0R0	U	P	2	TO	RCT	No	Yes	2	62	1	1
15	67	M	Oropharynx	pT2pN1Pv1M0R0	U	AB	0	TO	RCT	No	Yes	2	86	0	0
16	63	M	Oropharynx	pT2pN0M0R1	U	AB	0	TM	RCT	No	Yes	1	91	0	0
17	56	M	Oral cavity	pT1pN0M0R0	U	AB	0	TO	0	No	No	2	83	0	0
18	73	F	Oral cavity	pT1pN2bM0R0	U	AB	0	TO	0	No	Yes	2	79	0	0
19	66	F	Oral cavity	pT4apN0M0R0	B	AB	3	TO	RT	Yes	Yes	2	86	1	0
20	71	M	Oropharynx	pT2pN0M0R1	U	AB	0	TM	RT	Yes	Yes	1	93	0	0

^a^*U *unilatéral; *B* bilateral

^b^*AB*  antebrachial; *ALT*  anterolateral tigh; *P*  peroneal

^c^Stade following the Clavian-Dindo classification

^d^*TO*  transoral; *TM*  transmaxillar

^e^*RT*  radiotherapy; *RCT*  radiochemotherapy

^f^Closed margins

**Table 2:** Surgery results

Parameter	Value
Follow-up (range)	26.5 (7–29) months
Surgery	
U/B^a^	15 (75%)/5 (5%)
AB flap/ALT flap/P flap^b^	15 (75%) 2 (10%)/3 (15%)
TO/TM approach^c^	12 (60%)/8 (40%)
Tracheostomy^*^	18 (90%)^*^
Histological results	
R0/R1	18 (90%)/2 (10%)
Complications	
Total	11 (55%)
Grade II/Grade III^d^	2 (10%)/9 (45%)
Adjuvant treatments	
RT/RCT^e^	5 (25%)/7 (35%)
Gastrostomy required	4 (20%)

* One patient had a previous tracheostomy

^a^*U* unilatéral; *B* bilateral

^*b*^*AB* antebrachial; *ALT* anterolateral tigh; *P* peroneal

^c^Stade following the Clavian-Dindo classification

^*d*^*TO* transoral; *TM* transmaxillar

^*e*^*RT* radiotherapy; *RCT* radiochemotherapy

Median follow-up was 26.5 months. We performed 15 unilateral reconstructions (75%) and 5 bilateral reconstructions (25%). We used the transoral approach for 12 patients (60%) and the transmaxillary approach for eight patients (40%). Fifteen patients received an antebrachial flap (75%), two patients had an anterolateral thigh flap (10%), 3 had a peroneal flap (15%). Each patient with bilateral reconstruction underwent an antebrachial flap.

After surgery, definitive histological analysis showed no residual tumour (R0) for eighteen patients (90%) and microscopic residual tumour (R1) for two patients (10%). Four (20%) were R0 but with closed margins.

Eleven patients (55%) presented complications, among them two (10% of the total) grade II, and nine (45% of the total) grade III. Grade II complications were one donor site infection and one with cervical emphysema. Both showed good evolution after antibiotherapy. Grade III complications required revision surgery under general anaesthesia: three flap dehiscence were reinforced during a simple endoscopy with a transoral approach, as well as one oro-pharyngeal fistula. Two patients needed an anastomose revision for a venous kinking/thrombosis of the flap anastomosis. After a peroneal flap, one patient presented a necrosis of the skin graft covering the leg; a new partial thickness graft had to be taken and grafted. One antebrachial flap necrosed and had to be replaced totally with a new contralateral antebrachial flap. Finally, one patient with double antiplatelet therapy presented a mild hematemesis and underwent revision surgery for cauterization.

Two patients (10%) had previously received radiochemotherapy and chemotherapy for a pulmonary carcinoma and a laryngeal tumour.

After surgery, five patients (25%) required adjuvant radiotherapy and seven (35%) adjuvant radiochemotherapy. Patients with small tumors received radiochemotherapy in case of closed margins, perineural infriltration or perivascular infiltration. The surgical complications were managed in time and didn’t delay the adjuvant treatments.

Four patients (20%) required a gastrostomy, among which three had a unilateral flap and one a bilateral flap.

Eighteen tracheostomies were performed. For one patient, were realized at the beginning of the surgery that the flap would be relatively thin and small and that he would not need any tracheotomy. Another patient had a previous laryngectomy. The median time for closure was 14 days, considering that five patients still had their tracheostomy at the time of evaluation.

The median MDADI score was 89, and the mode was 93. A Fisher test shows a significant improvement of MDADI scores for unilateral vs bilateral reconstructions (*p* = 0.03).

**Fig. 4:**
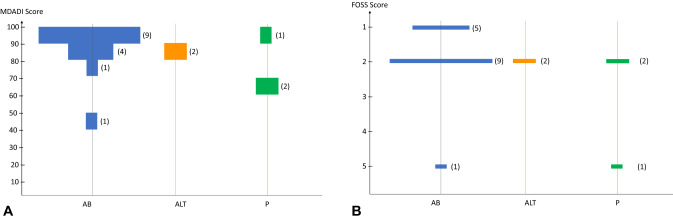
Violin plots, depending on the flap type (*AB* antebrachial; *ALT* anterolateral tight; *P* peroneal). **A** MDADI (A) score. **B** FOSS (B) score

The median FOSS score was 2, and the mode was 2. Fisher tests revealed no significant association with any type of surgery. Results of the FOSS score and MDADI scores are represented in Fig. 4. Functional outcomes are shown in Table 3.

**Table 3:** Functional results

MDADI score	
Median/Mode	89/93
U flap vs. B flap^a^	*p* = 0,031,908,247
AB vs. ALT and P^b^	*p* = 0,883,954,843
FOSS score	
Median/Mode	2/2
U flap vs. B flap^a^	*p* = 0,356,489,819
AB vs. ALT and P^b^	*p* = 0,356,489,819
Nasal regurgitation	7 (35%)
Tracheal aspiration	3 (15%)

*ALT* anterolateral tigh; *P* perone

^a^*U *unilatéral, *B* bilateral

^*b*^*AB* antebrachial; *ALT* anterolateral tigh, *P* peroneal

Seven (35%) patients complained of nasal regurgitation, regardless of the surgery they underwent.

Three (15%) reported episodic laryngeal aspiration; each had a peroneal flap.

Adjuvant treatment (radiotherapy or radiochemotherapy) did not have a significant effect on the MDADI and FOSS scores.

## Discussion

Soft palate tumours represent 15% of oropharyngeal tumours [6]. Until the 1990s, treatment was mainly surgical, and reconstruction was performed with locoregional flaps. For large tumours, functional outcomes were poor. In the 2000s, the arrival of new chemotherapy and radiotherapy techniques lead to place surgery as a second-line treatment.

Since then, surgery has evolved and combines reconstructions with implants and prostheses.

Today, free flaps dominate the reconstructive field. While maintaining the best oncologic outcome, it aims to restore the best functional result.

Nowadays, there are many surgical techniques. Some authors tried to establish a management standardization [1, 2] based on the expected defect size, but the precise surgical protocol remains depending on the surgeon’s habits and the patient’s preferences.

Many authors agree to recommend closing minor defects primarily. Locoregional flaps such as palatal island flap or buccinator myomucosal island flap have good functional results [7]. However, they have limited applications: they are restricted to small tumours, and they cannot be used in patients with a history of previous oropharyngeal radiotherapy. Tissue structure is damaged, and blood vessels unusable for vascular anastomosis. In such cases, free flaps are preferable. They provide a long pedicle and anastomosis in a healthy tissue area that was not subjected to radiation therapy [2].

For a long time, prothesis and obturators have been an alternative. It is cheaper than the reconstructive option and allows easier clinical monitoring for cancer recurrence [7]. However, it is difficult to make an obturator that ensures perfect sealing. Several weeks after the surgery or the radiotherapy, the oral cavity and oropharynx morphology can evolve, and the obturators need to be adapted. If it is not managed correctly or fixed, the patient will complain about discomfort, malodor, and persistent velopharyngeal insufficiency with leakage and oronasal regurgitation [2].

Most authors recommend an obturator for patients with large, complex, or multiple subsite defects, patients at high risk of undergoing anaesthesia for the reconstruction, or patients with a low survival probability [1].

Our treatment strategy meets most of the author’s recommendations [1, 2]. We perform local flaps for minor defects and limit obturators to fragile patients or those who refuse a new surgery. For extensive defects or after radiotherapy, we always propose a reconstruction with a free flap.

Opposite to local flaps, free-flaps bring healthy tissues. That gives the advantage of better healing in irradiated patients but also represents a limitation: the tissue transferred is not mucosa and subsequently lacks the benefits of similar form and function. Besides, the graft can be bulky and leaves a significant secondary defect. The surgeon's challenge is, therefore, to build a thin but stable in time reconstruction.

Antebrachial (AB) flap is the most used free flap [1, 2]. With its thinness and pliable propriety, it meets many requirements for a satisfying reconstruction. It can line both the nasal and oropharyngeal sides of the defect by folding on itself or by using a skin graft for the backside. It brings an adequate posterior bulk to contact the oropharyngeal wall and is flexible enough to be stitched on the lateral pharyngeal wall and the tongue base. There is almost no limitation of defect size.

In our study, we present our experience in soft palate reconstruction with free flaps.

We preferred using AB flaps. Two patients had bilateral unfavourable Allen tests, suggesting potential vascular disorders. Both underwent surgical reconstruction with an ALT flap. ALT was thicker than AB and also presented the disadvantage of intraoral hair growing in male patients.

We also performed three osteocutaneous free flaps because of osseous tumoral involvement. We choose peroneal flap because of our maxillofacial habits. This flap brings a large skin paddle and a solid bone for further dental implantation. Radial osteocutaneous forearm free flap is also commonly used, with the advantage of a more mobile skin paddle but a more fragile bone [7].

For the in-setting, particular care was given to stitch the flap as caudally as possible under the tongue base to tract the flap with a light tension. We believe that it assured a better sealing between the oropharynx and nasopharynx while avoiding airway obstruction.

**Fig. 5:**
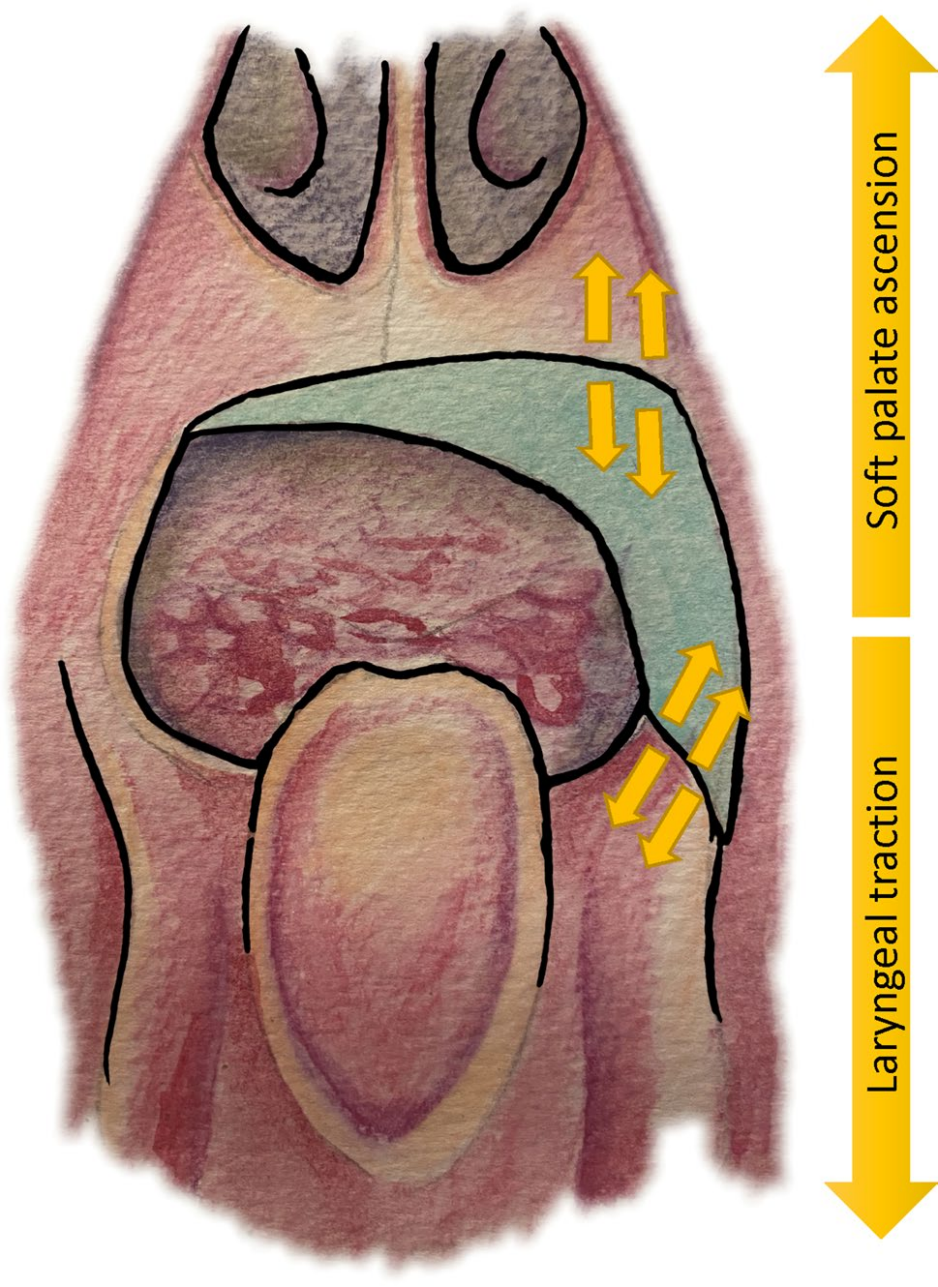
Posterior view. Areas of tension

However, it tenses the sutures and increases the risk of suture failure (Fig. 5). During swallowing, the soft palate tenses while the tongue base lowers. We recommend waiting 1 week before oral refeeding, but saliva swallowing will still stress the sutures. Three patients needed a new surgery for suture failure; each occurred on tension zones. It was easily re-sutured during endoscopic procedures.

Long-term (median follow-up: 26.5 months) evaluation shows encouraging results. Overall, MDADI and FOSS scores showed a good quality of life. Consistently, we get better results with unilateral than bilateral flaps, as it preserves sensibility and mobility.

Our study’s main limitation is the small sample of patients, limiting the statistical analysis. A prospective study would be more powerful. Moreover, it would allow comparing preoperative and postoperative scores. A postoperative functional endoscopic evaluation of swallowing could also give interesting objective results.

Finally, it would have been interested in considering the modification of the voice.

## Conclusion

Free-flap techniques to reconstruct soft palate defect after tumoral resection are challenging surgeries but provide good functional results. For large tumours, surgical resections and free-flap reconstruction should be proposed as a first-line treatment.

## Supplementary Information

Below is the link to the electronic supplementary material.

Supplementary file1 (DOCX 102 KB)

## Data Availability

Data are availabile.
