# Supplementary material for: Free flap for soft palate reconstruction: long-term functional evaluation of a new technique
Source: Eur Arch Otorhinolaryngol. 2021 Jun 2;279(3):1445–52. doi: 10.1007/s00405-021-06897-0 (PMC8897334; doi:10.1007/s00405-021-06897-0)
Supplement: Supplementary file 1 — Supplementary file1 (DOCX 102 KB) [file 405_2021_6897_MOESM1_ESM.docx]

**The M.D. Anderson Dysphagia Inventory**

This questionnaire asks for your views about your swallowing ability. This information will help us understand how you feel about swallowing. The following statements have been made by people who have problems with their swallowing. Some of the statements may apply to you. Please read each statement and circle the response which best reflects your experience in the past week :

| My swallowing ability limits my day-to-day activities  Strongly Agree Agree No Opinion Disagree Strongly Disagree |
| --- |
| E2. I am embarrassed by my eating habits.  Strongly Agree Agree No Opinion Disagree Strongly Disagree |
| F1. People have difficulty cooking for me.  Strongly Agree Agree No Opinion Disagree Strongly Disagree |
| P2. Swallowing is more difficult at the end of the day  Strongly Agree Agree No Opinion Disagree Strongly Disagree |
| E7. I do not feel self-conscious when I eat.  Strongly Agree Agree No Opinion Disagree Strongly Disagree |
| E4. I am upset by my swallowing problem.  Strongly Agree Agree No Opinion Disagree Strongly Disagree |
| P6. Swallowing takes great effort.  Strongly Agree Agree No Opinion Disagree Strongly Disagree |
| E5. I do not go out because of my swallowing problem.  Strongly Agree Agree No Opinion Disagree Strongly Disagree |
| F5. My swallowing difficulty has caused me to lose income.  Strongly Agree Agree No Opinion Disagree Strongly Disagree |
| P7. It takes me longer to eat because of my swallowing problem.  Strongly Agree Agree No Opinion Disagree Strongly Disagree |
| P3. People ask me, “Why can't you eat that?”  Strongly Agree Agree No Opinion Disagree Strongly Disagree |
| E3. Other people are irritated by my eating problem.  Strongly Agree Agree No Opinion Disagree Strongly Disagree |
| P8. I cough when I try to drink liquids.  Strongly Agree Agree No Opinion Disagree Strongly Disagree |
| F3. My swallowing problems limit my social and personal life.  Strongly Agree Agree No Opinion Disagree Strongly Disagree |
| F2. I feel free to go out to eat with my friends, neighbors, and relatives.  Strongly Agree Agree No Opinion Disagree Strongly Disagree |
| P5. I limit my food intake because of my swallowing difficulty.  Strongly Agree Agree No Opinion Disagree Strongly Disagree |
| P1. I cannot maintain my weight because of my swallowing problems.  Strongly Agree Agree No Opinion Disagree Strongly Disagree |
| E6. I have low self-esteem because of my swallowing problems.  Strongly Agree Agree No Opinion Disagree Strongly Disagree |
| P4. I feel that I am swallowing a huge amount of food.  Strongly Agree Agree No Opinion Disagree Strongly Disagree |
| F4. I feel excluded because of my eating habits.  Strongly Agree Agree No Opinion Disagree Strongly Disagree |


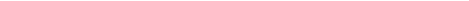


*Source:* Chen AY, Frankowski R, Bishop-Leone J, et al. The development and validation of a dysphagia-specific quality-of-life questionnaire for patients with head and neck cancer: the M. D. Anderson dysphagia inventory. Arch Otolaryngol Head Neck Surg. 2001;127:870–876. [PubMed]

**The Clavian-Dido classification :**


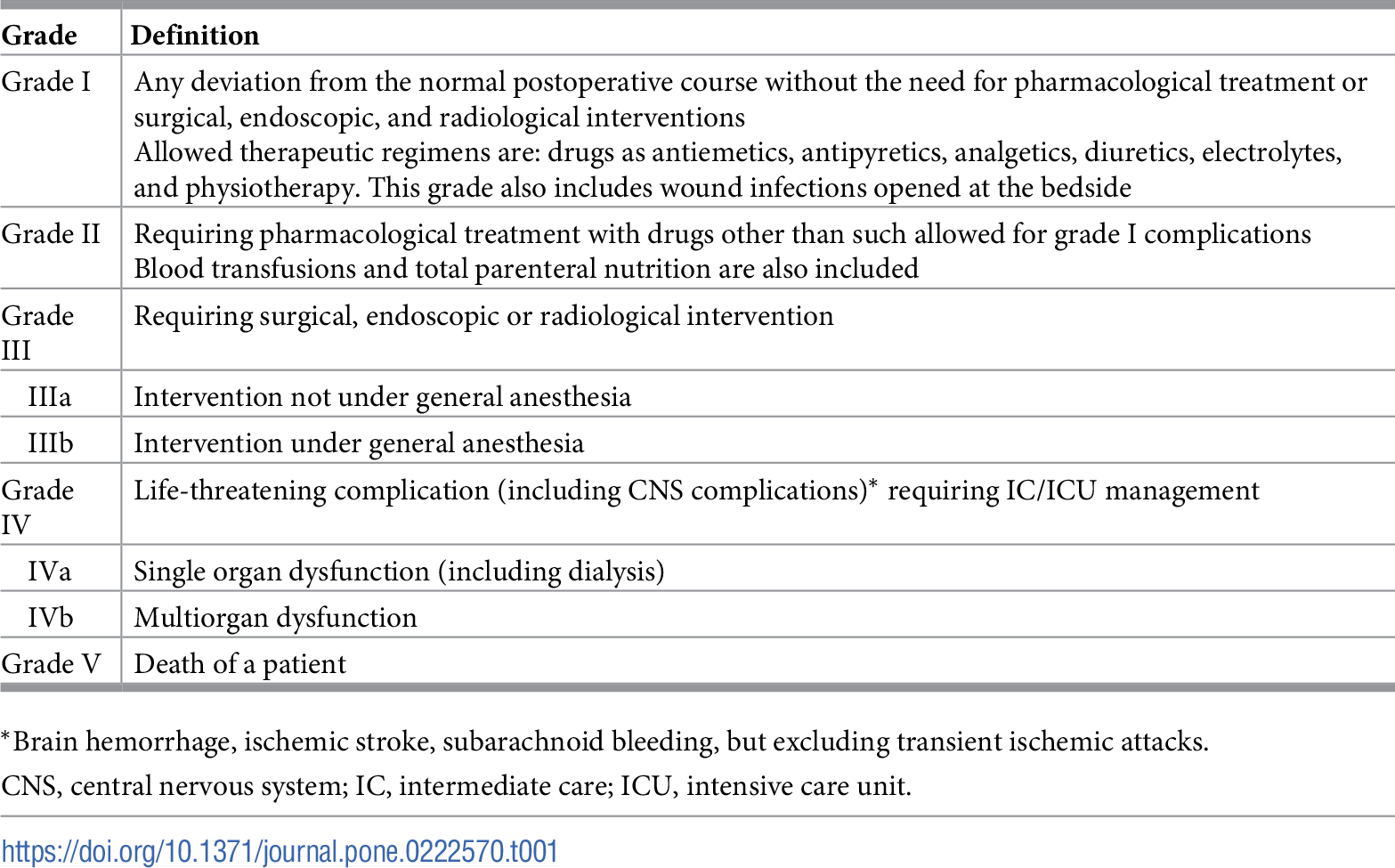


*Source :* Dindo D, Demartines N, Clavien PA. Classification of surgical complications: A new proposal with evaluation in a cohort of 6336 patients and results of a survey. Ann Surg. 2004;240(2):205–13. DOI: 10.1097/01.sla.0000133083.54934.ae

**FOSS score**

Stage Criteria

0 Normal physiologic function and asymptomatic

I Normal physiologic function (defined by no or minimal dietary modification, normal mealtime and no aspiration) but with episodic or daily symptoms of dysphagia such as reflux symptoms, globus, odynophagia, repetitive swallow, throat-clearing habit, difficulty chewing, minor oral incompetence (drooling), sensation that food sticks in throat or esophagus.

II Compensated abnormal function manifested by significant dietary modifications or prolonged mealtime (longer than one third).Weight is stable, cough is absent or occasional, aspiration is absent or occasional and mild (defined as aspiration cleared with a cough and limited to the subglottis, determined by barium swallow or video fiberoptic evaluation). Symptoms of stage may be present. Patients in this stage are stable in terms of nutrition and respiratory status, but swallowing behavior is modified.

III Decompensated abnormal function manifested by weight loss of 10% or less of bodyweight over 6 months due to dysphagia, or frequent cough, gagging, or aspiration during meals. Aspiration may be mild (as defined by stage II) or moderate (defined as silent or limited to the trachea). Patients in this stage are unstable in terms of nutrition or respiratory status. Pulmonary complications have not occurred, but the patient is at risk for them.

IV Severely decompensated abnormal function manifested by weight loss of more than 10% of body weight over 6 months due to dysphagia, or severe aspiration (defined as occurring below the trachea or any bronchopulmonary complications of aspiration). Non-oral feeding recommended for most (>50%) of nutrition. Patients in this stage are nearly complete swallowing failures and may safely swallow only under strictly defined conditions which do not meet their nutritional needs.

V Nonoral feeding for all nutrition. Patients in this stage are complete swallowing failures. They are different from stage IV in that they cannot swallow anything safely.

*Source* : Salassa JR. A functional outcome swallowing scale for staging oropharyngeal dysphagia. Dig Dis. 1999;17(4):230–4. DOI: 10.1159/000016941
